# Improved patient safety with a simplified operating room to pediatric intensive care unit handover tool (PATHQS)

**DOI:** 10.3389/fped.2024.1327381

**Published:** 2024-01-24

**Authors:** D. Subramonian, G. Krahn, J. Wlodarczak, L. Lamb, S. Malherbe, E. Skarsgard, M. Patel

**Affiliations:** ^1^ Division of Biochemical Diseases, BC Children’s Hospital, Department of Pediatrics, University of British Columbia, Vancouver, BC, Canada; ^2^ Division of Critical Care, BC Children’s Hospital, Department of Pediatrics, University of British Columbia, Vancouver, BC, Canada; ^3^ Office of Virtual Health, Provincial Health Services Authority, Vancouver, BC, Canada; ^4^ Division of General Surgery, BC Children’s Hospital, Department of Surgery, University of British Columbia, Vancouver, BC, Canada; ^5^ Division of Cardiac Anesthesia, BC Children’s Hospital, Department of Anesthesia, University of British Columbia, Vancouver, BC, Canada

**Keywords:** handover, PICU, safety, tool, OR, quality improvement

## Abstract

**Introduction:**

Patient handover is a crucial transition requiring a high level of coordination and communication. In the BC Children's Hospital (BCCH) pediatric intensive care unit (PICU), 10 adverse events stemming from issues that should have been addressed at the operating room (OR) to PICU handover were reported into the patient safety learning system (PSLS) within 1 year. We aimed to undertake a quality improvement project to increase adherence to a standardized OR to PICU handover process to 100% within a 6-month time frame. In doing so, the secondary aim was to reduce adverse events by 50% within the same 6-month period.

**Methods:**

The model for improvement and a Plan, Do, Study, Act method of quality improvement was used in this project. The adverse events were reviewed to identify root causes. The findings were reviewed by a multidisciplinary inter-departmental group comprised of members from surgery, anesthesia, and intensive care. Issues were batched into themes to address the most problematic parts of handover that were contributing to risk.

**Intervention:**

A bedside education campaign was initiated to familiarize the team with an existing handover standard. The project team then formulated a new simplified visual handover tool with the mnemonic “PATHQS” where each letter denoted a step addressing a theme that had been noted in the pre-intervention work as contributing to adverse events.

**Results:**

Adherence to standardized handover at 6 months improved from 69% to 92%. This improvement was sustained at 12 months and 3 years after the introduction of PATHQS. In addition, there were zero PSLS events relating to handover at 6 and 12 months, with only one filed by 36 months. Notably, staff self-reporting of safety concerns during handover reduced from 69% to 13% at 6 months and 0% at 3 years. The PATHQS tool created in this work also spread to six other units within the hospital as well as to one adult teaching hospital.

**Conclusion:**

A simplified handover tool built collaboratively between departments can improve the quality and adherence of OR to PICU handover and improve patient safety. Simplification makes it adaptable and applicable in many different healthcare settings.

## 1 Introduction

### 1.1 Problem description

The BC Children's Hospital (BCCH) pediatric intensive care unit (PICU) is a tertiary care critical care unit in a teaching hospital with 1,000 admissions per year and a full surgical program including cardiac surgery. Approximately half of the admissions to the PICU come directly from the OR. The hospital utilizes a system known as the patient safety learning system (PSLS) to report adverse events relating to patient care. These events are categorized by severity and reviewed to look for improvement opportunities. The BCCH PICU experienced 10 PSLS events over the course of 12 months that specifically stemmed from OR to PICU handover of patient care. These events were mostly near miss or minor harm events, but in a PICU environment, the potential for harm from these near miss events was significant given the latent threats that these errors posed. Issues included: intravenous lines (IV) that were believed to be infusing drugs but were actually clamped; patients who were emerging from anesthesia and needing active medical management while handover continued; patients not fully hooked to PICU monitors so changes in vital signs not visible to the wider team for the duration of handover; attending physicians variably present at handover; and key pieces of family information not being relayed to the PICU team resulting in staff and familial frustration with changing post-op cares plans. In addition to the PSLS events, there was one formal family complaint around poor translation of information from pre-operative to postoperative care of their child. There was a vital necessity for handover process (OR to PICU) evaluation and standardization or the unit risked a major harm event and distress to families.

### 1.2 Background

Patient handovers, or transitions of care between medical providers in hospitals, are high-risk times in regard to patient safety (1). In addition, postoperative handovers occur in dynamic environments where providers are multitasking and not always known to one another, enhancing the potential for medical errors and loss of information (2). The increasing interest in patient safety and quality of care has contributed to many proposed tools to improve OR to ICU handover over the past years (3–5). Few of these published tools reference adverse event metrics as an outcome measure (rather measure tool adherence alone), and there is a lack of evidence demonstrating long lasting change after implementation of these tools (6–8). This makes the impact of such tools difficult to interpret.

Despite the available knowledge around the benefits of employing handover tools and the existence of such a tool in the unit, the BCCH PICU with its large postoperative surgical population was not adhering to a standardized handover process, and there were adverse events as a result. It was postulated that re-invigorating an OR to PICU standard handover process would mitigate the challenges that were being reported and improve patient safety.

### 1.3 Rationale

Before implementing any changes, the project team set out to better define the problems that were leading to these PSLS events. From February to April 2018, a formal observation period was undertaken auditing gaps in surgical admissions from OR to PICU. In addition, the teams were invited to report any “general safety concerns” they perceived at the end of the handover. These general safety concerns were a subjective report of any issue that made the receiving team feel that the safety of their patient was compromised. This was collected anonymously as free text space at the end of the audit form so there would be psychological safety in raising any issue that was of concern to the team member.

During this pre-implementation period (February–April 2018), patterns in handover gaps were noted. First, the current handover tool was never utilized for any of the handovers. Second, the audits confirmed the common themes in the PSLS reports with site to source* being done improperly [*a process by which the anesthesia physician (MD) reviews every indwelling line and tube from the patient to the pump in the presence of the PICU nurse (RN) to confirm clamp position, drug name, concentration, patient weight and delivered dose. This serves as both a double check of drug dosing and delivery as well as informs the receiving nurse of which meds are running where and what lines are available. In the BCCH PICU, it is mandatory for nursing staff to perform a site to source upon taking over care of any new patient from any type of healthcare provider]. In addition, it was noted that not all team members were present and ready to receive handover, yet handover would begin; not all patients were hooked up to their monitors, and some patients were requiring ongoing medical management during handover. A concerning finding was that staff reported having a general “safety concern” of any description 69% of the time after receiving OR handover. In this regard, the teams reported that they did not always know the staff member handing over nor their role, making the comfort level for asking clarifying questions low leaving the team sometimes confused about postoperative care plans. Some other general safety concerns included worry around tangled IV lines, handover being done in siloes (MD to MD, RN to RN), lack of attending-level MD presence, physicians on their phones during handover, missing equipment, or poor communication in general around the status of the patient and the expected trajectory or any family issues of concern. There were also observed knowledge gaps around what constituted a complete site to source check.

**Figure 1:**
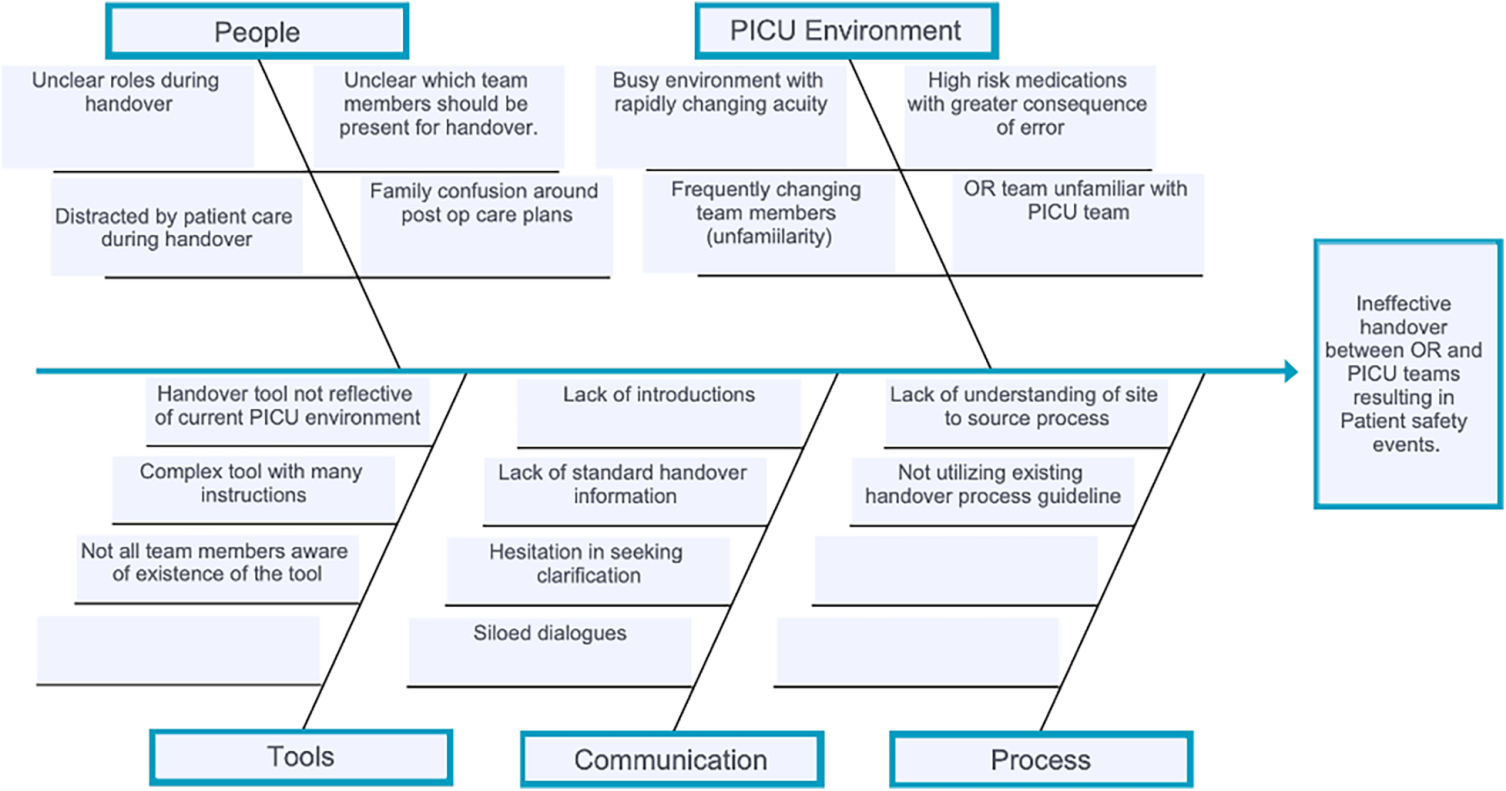
Cause and effect diagram.

All these types of issues were plausibly contributing to adverse events either directly or indirectly and needed mitigation. Utilizing cause and effect diagramming, the project team identified education gaps, communication gaps, inadequate tools, and environmental factors as contributing to the errors being reported (Figure 1).

### 1.4 Specific aim

A cross-departmental collaborative project team was created and set the aim to achieve 100% adherence to a standardized handover process from OR to PICU within 6 months. In doing so, it was hypothesized that we would achieve a secondary aim of reducing PSLS events related to handover by at least 50% in the study period thus improving safety for this entire population of patients.

## 2 Method

### 2.1 Project context

BC Children's Hospital is a tertiary care children's hospital with a population catchment of 5 million. It offers all levels of subspecialty care and is a teaching hospital with a pediatrics residency program as well as multiple fellowship programs including surgery, anesthesia, and critical care. PICU cares for critically ill children of all medical, cardiac, and non-cardiac surgical subspecialties. It consists of 18 beds with 1:1 nursing for nearly all postoperative patients. Average admissions in PICU are 1,000 per year for the last 5 years—50% from the OR including cardiac surgical patients. During our project period, June 2018–Jan 2019, there were 465 admissions to PICU with 204 transfers from the OR to PICU.

When receiving a patient from the OR, the previous unit practice was for a verbal handover to take place between multidisciplinary, cross-departmental teams. This handover included the PICU team, anesthesia team, and occasionally a representative from the surgical teams. There was no standard in whether the representative from a respective team was house staff or consultant level and no set time for OR nurses to share details they needed to include. A handover standard and tool that had been established 10 years prior however had complex guidelines, was time consuming, and did not facilitate shared understanding of the patient. In interviewing staff regarding its use, they reported that it had virtually been abandoned in practice due to the level of detail and lack of practicality. Thus, handovers were being conducted *ad hoc*, at the discretion of the team who were present for any given handover.

### 2.2 Intervention strategies

After the pre-implementation period identified gaps and a lack of adherence to the current handover standard, the improvement period began with the project team first making modifications to the old tool to simplify some of the language and then undertook a re-education campaign for all the stakeholders regarding the use of this tool. Particular emphasis was made on the themes that had been noted from the PSLSs and audits. This included addressing that all team members needed to be present prior to commencing handover, completing a proper site to source, and making sure the patient was stable before commencing handover. Clinical nurse educators (CNE) in the ICU did the handover tool and site to source re-education with bedside staff daily over a 2-week period. This was done 1:1 from bedside to bedside within the PICU itself. The project team members undertook small group sessions with stakeholders from surgery, anesthesia, and PICU to educate on the use of the tool and the highlighted concerns. The physician staff were notified at a leadership level of the expectation for senior MD presence for all handovers for PICU, anesthesia, and surgery teams.

After the education phase, another audit was undertaken with 19 OR to PICU handovers directly observed with the expectation that results would have improved. It was noted that the re-education had marginal impact on improving the quality of handover. The complete team being present improved from 69% to 78%; however, site to source checks actually dropped from 81% to 73%, the patients were only stable prior to commencing handover 73% of the time (virtually unchanged), and there were still perceived patient general safety concerns from the team in 40% of handovers.

**Figure 2:**
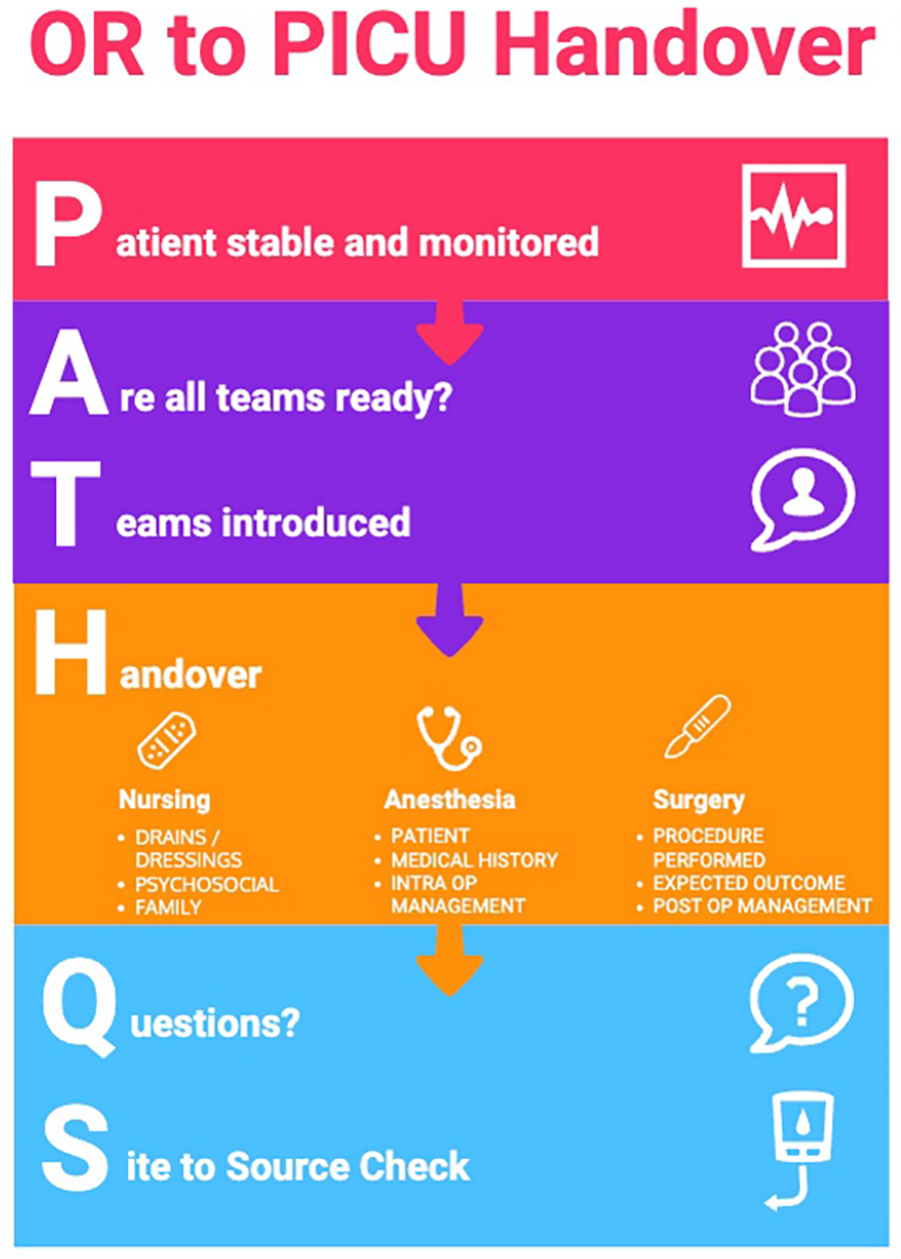
PATHQS handover standard incorporating the frequently missed elements.

After finding that the same issues noted in the pre-implementation phase persisted after the re-education change idea, the project team decided upon a second change idea. This was to address the feedback about the cumbersome handover tool that was in use by implementing a new tool that would specifically address the pre-implementation themes that had been documented. A new handover standard was created and the team undertook several Plan Do Study Act cycles (PDSAs) to refine the proposed handover tool. Of note, this was not a checklist *per se*, rather a memory aide that distilled down to the important elements of handover that seemed to be repeatedly resulting in gaps in patient safety. The project team identified solutions to gaps identified in our handover process and captured them in a bright new simplified standard using the mnemonic PATHQs (Figure 2):

(1) **P**atient stable and monitored
(2) **A**ll teams present
(3) **T**eams introduced
(4) **H**andover in a set order of speakers across teams
(5) **Q**uestions?
(6) **S**ite to Source

**Figure 3:**
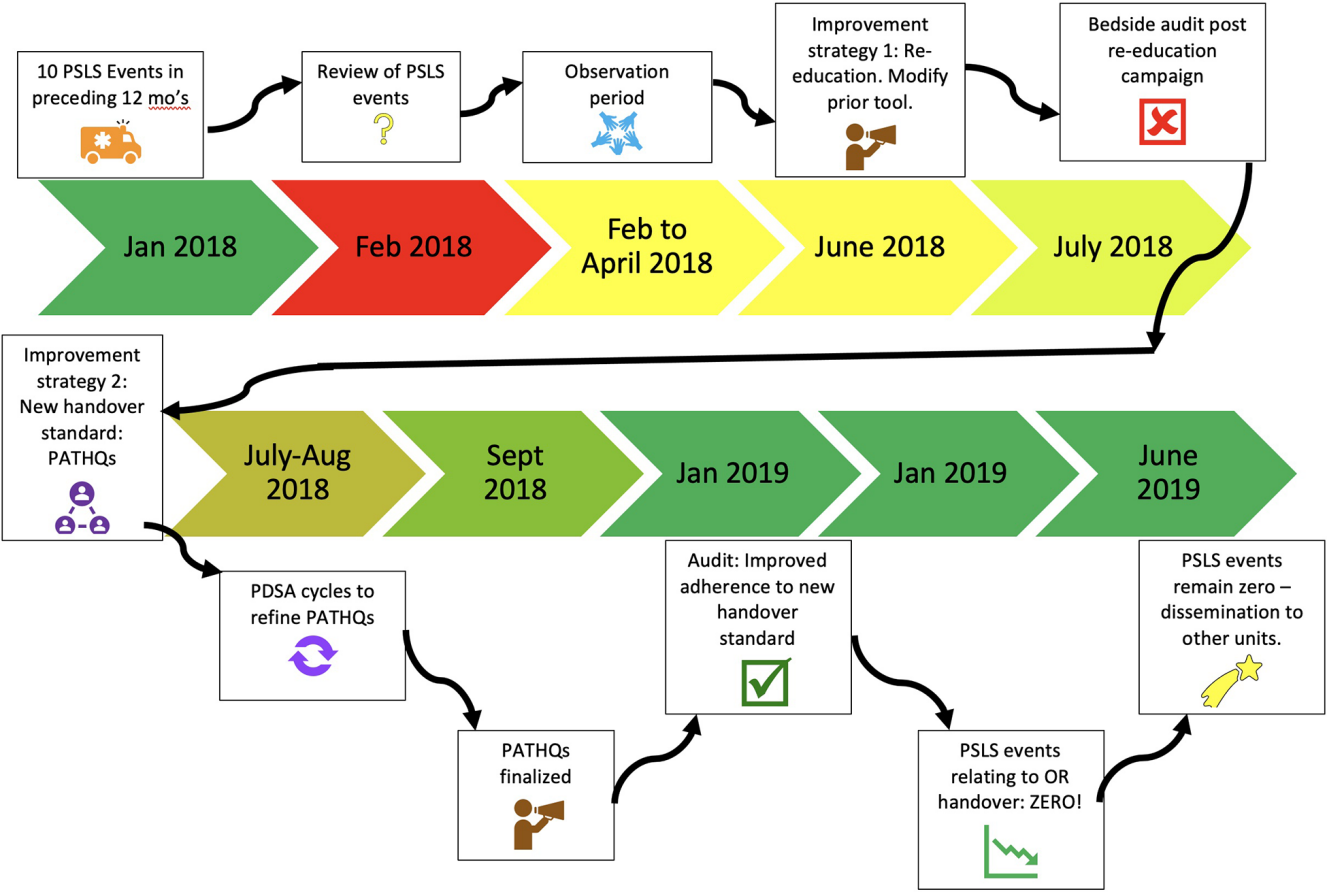
Timeline of handover improvement project.

The final tool emphasized six distinct elements of conduct and communication, was an interactive process, and involved a comprehensive review of the intraoperative and anticipated postoperative course. The wider team was educated on the new PATHQS tool utilizing a number of strategies including email communication, small group sessions with stakeholders, 1:1 communication and education at the bedside within the PICU, and then physical presence in the PICU of project team members during the initial roll out of the PATHQS. The steps and timeline of the work can be seen in Figure 3.

## 3 Results

The PATHQS was successfully rolled out as a standardized handover process in June 2018. This standard handover process was successfully utilized at 92% of audited handovers 6 months after implementation, improved from 69% in the pre-implementation phase, however, falling just short of the 100% target set in the primary aim. It is notable that its usage grew over time and at both the 12- and 36-month mark, the tool was utilized in 100% of the audited handovers (Table 1).

**Table 1:** Results of improvement strategies.

Rounding target (handovers observed)	Pre-implementation phase February–April 2018 (16)	Improvement phase June to December 2018		Post-improvement phase		
		Post-education intervention (19)	PATHQS intervention	6 months (24)	12 months (5)	36 months (12)
Stable vitals, patient ready for handover	69%	73%	*P*atient stable	92%	100%	100%
Personnel present and ready for handover	69%	78%	*A*ll teams ready	83%	80%	83%
Team introductions	n/a	n/a	*T*eam introductions	46%	0%	83%
Handover by all services	69%	94%	*H*andover by all services	92%	100%	100%
Opportunity for questions	n/a	n/a	Opportunity for *Q*uestions	67%	40%	83%
Site to source	81%	73%	*S*ite to source	96%	100%	100%
Patient safety concern reported after completion of handover	69%	40%	Patient safety concern noted	13%	20%	0
PSLS #	10 in 1 year prior		PSLS	0	0	1

**Figure 4:**
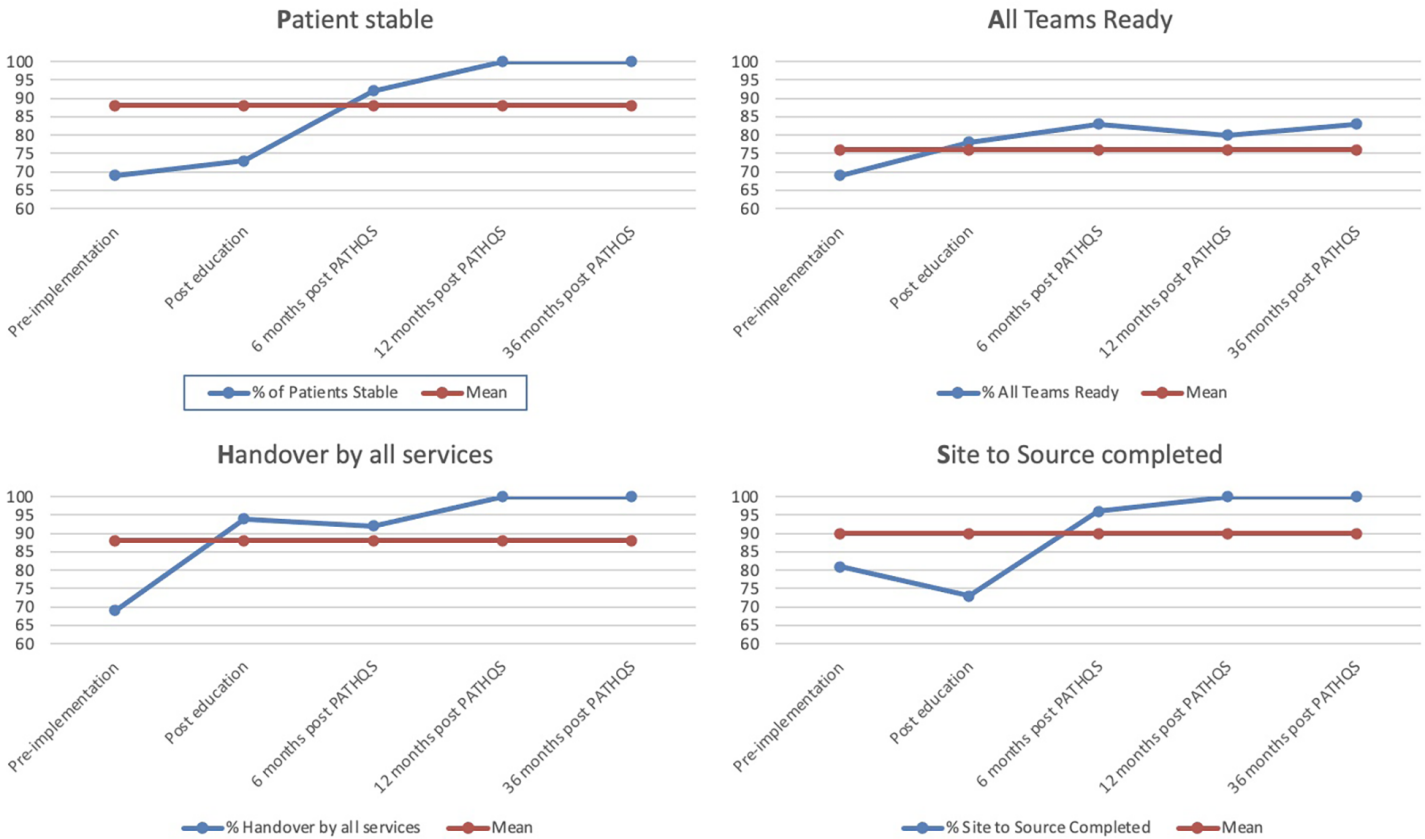
Results of handover improvement.

Of note, in the 6-month timeframe, patients being stable and monitored increased from 69% to 92%, site to source checks went from 81% to 96%, all team members ready went from 69% to 83%, and safety concerns reduced from 69% to 13%. The improvement from pre-implementation to 36 months (Figure 4) was even more impressive demonstrating that over a *36-month* timeframe,

- **P**atients being stable and monitored prior to handover improved from 69% to 100%,
- **A**ll team members being ready to receive handover improved from 69% to 83%,
- **T**eam introductions improved from not happening at all to 83%,
- **H**andover standard adherence improved from 69% to 100%,
- **Q**uestions invited: went from not happening at all to 83%, and
- **S**ite to source adherence improved from 81% to 100%.

**Figure 5:**
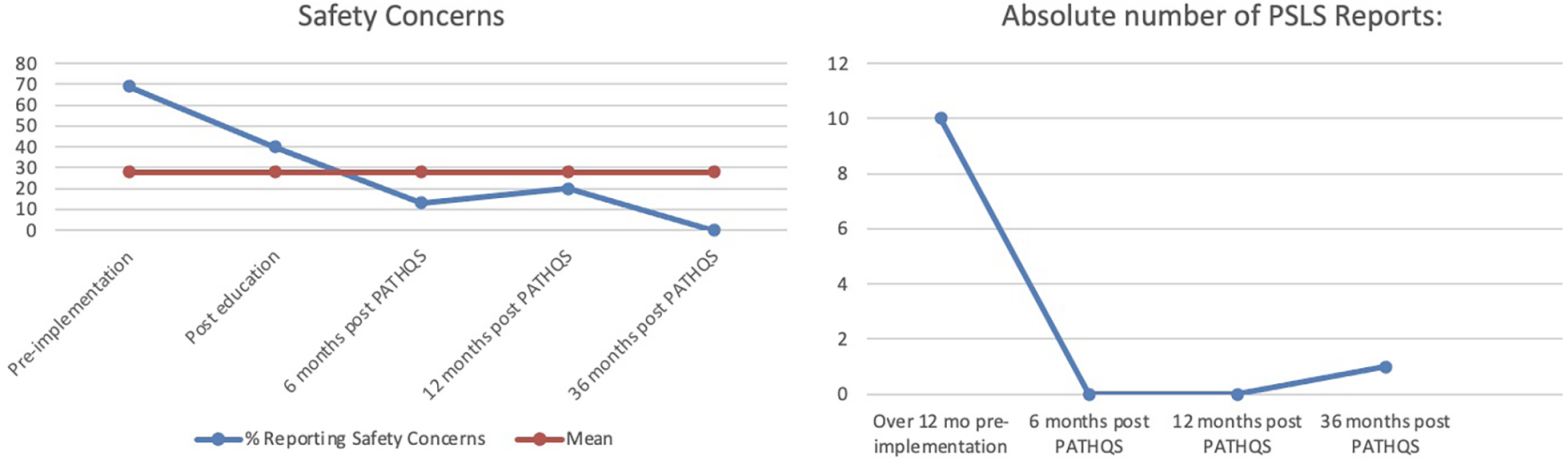
Reduced safety concerns and PSLS events.

Regarding the secondary aim of reducing adverse events by 50%, there were zero PSLS events filed relating to handover at 6 months, persisting to 12 months after the improvement strategy implementation. At the 36-month mark, there had been only a single PSLS filed related to the handover items on the PATHQS (Figure 5). Safety concerns qualitatively reported by the staff after a handover reduced from 69% at pre-implementation to 0% at the 36-month mark.

After the results were collated, the tool was adapted successfully to six other settings within the hospital and adopted by a local adult facility as well (Supplementary Material 1).

## 4 Discussion

These results are impressive and should have impact on how teams approach handover improvement. The use of systematized communication tools and checklists have demonstrated benefits in meeting performance and safety standards and reducing errors in industries such as aviation or product manufacturing, where the welfare of a human being is at risk (9). In healthcare settings, several studies have shown improvement in the process of information transfer by introducing handoff checklists in the evaluation and therapy of critically ill patients, such as initial assessment of trauma patients or in cardiac care situations (10–14). Though interest and application of such tools has become widespread in the handover of critically ill patients, there is limited research done that connect the use of these tools to a reduction in adverse events during the handover process from OR to PICU (15–19). Various studies have shown that communication failure such as inadequate information transfer is a usual concern for patient safety in most types of handovers (20–22), and various reviews have reported the effectiveness of standardizing the handoff tools to improve the information transfer and patient care (23–26). Chenault et al. and Riley et al. have demonstrated sustainability of protocolized handover process in pediatric patients transferred to intensive care after cardiac surgery (27, 28) The current handover improvement has been demonstrated to be easy for teams to adopt, easy to adapt to other handover environments, sustainable, and effective in reducing adverse events stemming from handover. The PATHQS is unique in its ability to bridge all of these issues in a single tool.

The current study is novel in that it specifically measured patient safety outcomes relating to a handover process, not simply adherence to a process. In regards to the process, the current quality improvement study exhibits excellent uptake of a proposed tool and thereafter an actual reduction in reported adverse events lasting 12 and 36 months after implementation. This body of work has demonstrated cross-departmental and cross-discipline effectiveness and uptake of a handover tool that not only created a standard process but also improved patient safety.

Education is the long-standing backbone of many attempts to improve patient care in medicine, but this work demonstrates that education alone is not necessarily sufficient when embarking on change in human behavior. Blyth et al. have recommended that additional measures than education alone are needed to have an impact on clinical handover improvements (29). The current study reinforces this notion as after a fulsome re-education campaign, we observed an unsteady improvement in handover concerns. Of interest is that despite the education around site to source, the audit demonstrated worse adherence to the site to source standard (81%–73%). We believe this is because the education campaign in fact reminded teams as to what constituted a proper site to source check likely resulting in the audit period reflecting a more accurate measure of the status quo (73%) as opposed to the pre-implementation period when staff were accepting sub-standard site to source checks (81%). Education, however, did not improve any of the other noted gaps in the handover process sufficiently. The tool was still cumbersome and that had not been addressed once again supporting that education alone is not sufficient to improve handover processes and that standardizing processes is a stronger method of making lasting change.

Considering these observations and taking into account our team members’ need for a less cumbersome tool to address the issues without additional documentation, we came up with six simplified elements in creating a standard for OR to PICU handover ensuring that essential components were included. The use of a mnemonic aided in recollection of the essential steps and the simplicity enhanced adoption and acceptability within our group. In addition, the observed safety improvement was noted despite the absence of adopting a formal checklist.

One of the encountered barriers to implementation was a concern that time to handover would increase with the PATHQS process. Previous studies have been done to better understand the handoff communication process and improve the quality and safety of care of postoperative cardiac patients in a pediatric cardiac ICU setup (30–33). Zavalkoff et al. and Joy et al. showed improvement in the critical information transfer without change in the duration of handoff in postoperative cardiac patients (32, 33). Though we were unable to time handovers in the current study, this would have been an important balancing measure and was recommended to our NICU colleagues as an area to explore further during their own implementation of the PATHQS. Costaldo et al. in the BC Women's Hospital NICU adapted and then adopted the PATHQS. Using time to handover as a balancing measure, they found an actual reduction in handover time for OR to NICU handovers utilizing PATHQS [Costaldo M (2023), email communication, Nov 21]). This adds to the body of evidence that the duration of handover does not necessarily increase with the implementation of a handover tool.

An interesting observation in our study was that after the initial success in rolling out this process, at the 12-month mark, there was actually a reduction in team introductions and inviting questions. As feedback, we were informed that these two steps felt awkward, and therefore over time, the teams were shying away from implementing these elements of the process. Though implementing the PATHQS created a structure for these steps to take place, it had not addressed the human factors that led to discomfort in following the more interactive parts of the tool. We believe the subsequent improvement in those domains seen at 36 months was due to “spread” within our facility. With other units adapting and adopting the PATHQS, the anesthesia team became very familiar with its use and implementation. This led to the anesthesia team driving the elements of the PATHQS creating psychological safety for the PICU team to engage in introductions and to ask questions allowing both teams to more comfortably adopt the tool fully.

To our knowledge, this project is first of its kind to use the same handover tool uniformly across a mixed medical and surgical PICU with demonstrated improvement in patient safety. There was also sustainability with this simplified tool as it has been used to successfully reduce adverse events for 36 months and counting. It is also unique in its uptake across programs and hospitals traversing pediatric, neonatal, and adult units. This simplicity and cross-applicability make this a powerful tool in improving handover in medicine across the spectrum.

### 4.1 Limitations

One of the limitations of this study is the possibility of the PSLS reporting fatigue over time. However, the BCCH PICU has very high rates of reporting overall for all adverse events encountered in the PICU, and these rates did not change in the study period. In addition, given the cross-departmental awareness of this work and heightened awareness of the standards, teams were encouraged to report gaps as we progressed through this work, standing to reason that the reduction in PSLS reports related to handover was reflective of a true reduction in adverse events. Similarly, the improvement in “general safety concerns” and PSLS rate over 36 months likely reflects true improvement as these concerns were reported anonymously, and there was no change in culture or environment that would have made reporting less likely. If anything, awareness around this handover improvement project created heightened awareness among the team to report any safety concern related to OR to PICU handover, standing to reason concerns would be more likely to be reported. This makes the resulting safety improvement likely truly reflective of improved patient safety.

Families of patients were not included in the project and the onset of the COVID-19 pandemic thwarted future plans of including families on handover. Future projects should include families from the outset. Though it may have been possible that the 6-month improvement was due to a Hawthorne effect in this study, this tool in 1- and 3-year observations has shown good sustainability. The use of random sampling and not collecting data on all OR to PICU handovers during the time period may have led to a selection bias, but it is unclear this would have affected the results. The handovers happening in the after-hour times or on weekends and holidays usually have less participants and were not included in this project. In spite of these limitations, this work displays that development of such a tool which is locally acceptable and sustainable in a multidisciplinary PICU, where a large number of staff are involved, is achievable.

Future studies incorporating larger numbers of observations, longer-term follow-up, and covering wider aspects of handover quality (e.g., time pressure and handover preparedness) with a positive effect on patient safety outcomes could be of additional value.

## 5 Conclusion

This project demonstrated that a simplified handover standard considering the local culture can be built collaboratively, and it can improve patient safety during OR to PICU handover, which is also sustainable over time. In addition, the PATHQS tool served as a model for interdisciplinary handovers in various other clinical environments demonstrating its agility and applicability in a variety of settings.

## Data Availability

The original contributions presented in the study are included in the article/**Supplementary Material**, further inquiries can be directed to the corresponding author.

## References

[1] Siemsen IMD Michaelsen L Nielsen J Østergaard D Andersen HB. Patient handover involves numerous safety risks. Ugeskr Laeger. (2011) 173:1412–6. 21586244

[2] Møller TP Madsen MD Fuhrmann L Østergaard D. Postoperative handover. Eur J Anaesthesiol. (2013) 30(5):229–42. 10.1097/EJA.0b013e32835d8520 23492933

[3] Petrovic MA Aboumatar H Baumgartner WA Ulatowski JA Moyer J Chang TY Pilot implementation of a perioperative protocol to guide operating room-to-intensive care unit patient handoffs. J Cardiothorac Vasc Anesth. (2012) 26:11–6. 10.1053/j.jvca.2011.07.009 21889365

[4] Catchpole KR de Leval MR McEwan A Pigott N Elliott MJ McQuillan A Patient handover from surgery to intensive care: using formula 1 pit-stop and aviation models to improve safety and quality. Paediatr Anaesth. (2007) 17:470–8. 10.1111/j.1460-9592.2006.02239.x 17474955

[5] Mueller SK Call SA McDonald FS Halvorsen AJ Schnipper JL Hicks LS. Impact of resident workload and handoff training on patient outcomes. Am J Med. (2012) 125:104–10. 10.1016/j.amjmed.2011.09.005 22195534

[6] Patterson ES Wears RL. Patient handoffs: standardized and reliable measurement tools remain elusive. Jt Comm J Qual Patient Saf. (2010) 36:52–61. 10.1016/s1553-7250(10)36011-9 20180437

[7] Aase K Vasshus HA Meling M. Safety in the transition between shifts: a quantitative study within healthcare. In: Aven T Vinnem JE, editors. Risk, Reliability and Social Safety. London: Taylor & Francis (2007). p. 1209–15.

[8] Lawrence RH Tomolo AM Garlisi AP Aron DC. Conceptualizing handover strategies at change of shift in the emergency department: a grounded theory study. BMC Health Serv Res. (2008) 8:256. 10.1186/1472-6963-8-256 19087251 PMC2640383

[9] Hales BM Pronovost PJ. The checklist—a tool for error management and performance improvement. J Crit Care. (2006) 21:231–5. 10.1016/j.jcrc.2006.06.002 16990087

[10] Committee on Trauma, American College of Surgeons. Advanced Trauma Life Support Course. 9th ed. Chicago, IL: American College of Surgeons (2012).

[11] Deakin CD Nolan JP Soar J Sunde K Koster RW Smith GB European Resuscitation Council Guidelines for Resuscitation 2010. Section 4. Adult advanced life support. Resuscitation. (2010) 81:1305–52. 10.1016/j.resuscitation.2010.08.017 20956049

[12] Kaufman J Twite M Barrett C Peyton C Koehler J Rannie M A handoff protocol from the cardiovascular operating room to cardiac ICU is associated with improvements in care beyond the immediate postoperative period. Jt Comm J Qual Patient Saf*.* (2013) 39:306–11. 10.1016/s1553-7250(13)39043-6 23888640

[13] Stahl K Palileo A Schulman CI Wilson K Augenstein J Kiffin C McKenney M. Enhancing patient safety in the trauma/surgical intensive care unit. J Trauma. 2009; 67(3):430–3. discussion 433–5. 10.1097/TA.0b013e3181acbe75 19741381

[14] Zavalkoff SR Razack SI Lavoie J Dancea AB. Handover after pediatric heart surgery: a simple tool improves information exchange. Pediatr Crit Care Med. (2011) 12(3):309–13. 10.1097/PCC.0b013e3181fe27b6 20975613

[15] Riesenberg LA Leitzsch J Little BW. Systematic review of handoff mnemonics literature. Am J Med Qual. (2019) 34(5):446–54. 10.1177/1062860619873200 31479296

[16] Davis J Roach C Elliott C Mardis M Justice EM Riesenberg LA. Feedback and assessment tools for handoffs: a systematic review. J Grad Med Educ. (2017) 9(1):18–32. 10.4300/JGME-D-16-00168.1 28261391 PMC5319625

[17] Breuer RK Taicher B Turner DA Cheifetz IM Rehder KJ. Standardizing postoperative PICU handovers improves handover metrics and patient outcomes. Pediatr Crit Care Med. (2015) 16(3):256–63. 10.1097/PCC.0000000000000343 25607744

[18] Malenka EC Nett ST Fussell M Braga MS. Improving handoffs between operating room and pediatric intensive care teams: before and after study. Pediatr Qual Saf. (2018) 3(5):e101. 10.1097/pq9.0000000000000101 30584628 PMC6221591

[19] Kamath SS Helmers L Otto A Kirk D Erdahl J Wayling B. Operating room to pediatric intensive care unit handoff: improving communication and team relations while driving process improvement. J Anesth Clin Care. (2016) 3:1–7. 10.24966/ACC-8879/100017

[20] Horwitz LI Moin T Krumholz HM Wang L Bradley EH. Consequences of inadequate sign-out for patient care. Arch Intern Med. (2008) 168:1755–60. 10.1001/archinte.168.16.1755 18779462

[21] Horwitz LI Meredith T Schuur JD Shah NR Kulkarni RG Jenq GY. Dropping the baton: a qualitative analysis of failures during the transition from emergency department to inpatient care. Ann Emerg Med. (2009) 53:701–10. 10.1016/j.annemergmed.2008.05.007 18555560

[22] Horwitz LI Moin T Krumholz HM Wang L Bradley EH. What are covering doctors told about their patients? Analysis of sign-out among internal medicine house staff. Qual Saf Health Care. (2009) 18:248–55. 10.1136/qshc.2008.028654 19651926 PMC2722040

[23] Rosenthal JL Doiron R Haynes SC Daniels B Li S-TT. The effectiveness of standardized handoff tool interventions during inter- and intra-facility care transitions on patient-related outcomes: a systematic review. Am J Med Qual. (2018) 33(2):193–206. 10.1177/1062860617708244 28467104

[24] Kripalani S LeFevre F Phillips CO Williams MV Basaviah P Baker DW. Deficits in communication and information transfer between hospital-based and primary care physicians: implications for patient safety and continuity of care. JAMA. (2007) 297:831–41. 10.1001/jama.297.8.831 17327525

[25] Foster S Manser T. The effects of patient handoff characteristics on subsequent care: a systematic review and areas for future research. Acad Med. (2012) 87:1105–24. 10.1097/ACM.0b013e31825cfa69 22722354

[26] Li P Ali S Tang C Ghali WA Stelfox HT. Review of computerized physician handoff tools for improving the quality of patient care. J Hosp Med. (2013) 8:456–63. 10.1002/jhm.1988 23169534

[27] Chenault K Moga MA Shin M Petersen E Backer C Jr DOG Sustainability of protocolized handover of pediatric cardiac surgery patients to the intensive care unit. Paediatr Anaesth. (2016) 26(5):488–94. 10.1111/pan.12878 26997082

[28] Riley CM Merritt AD Mize JM Schuette JJ Berger JT. Assuring sustainable gains in interdisciplinary performance improvement: creating a shared mental model during operating room to cardiac ICU handoff. Pediatr Crit Care Med. (2017) 18(9):863–8. 10.1097/PCC.0000000000001231 28654551

[29] Blyth C Bost N Shiels S. Impact of an education session on clinical handover between medical shifts in an emergency department: a pilot study. Emerg Med Australas. (2017) 29:336–41. 10.1111/1742-6723.12717 28004506

[30] Chen JG Wright MC Smith PB Jaggers J Mistry KP. Adaptation of a postoperative handoff communication process for children with heart disease: a quantitative study. Am J Med Qual. (2011) 26:380–6. 10.1177/1062860610394342 21701043 PMC3261576

[31] Mistry KP Jaggers J Lodge AJ Alton M Mericle JM Frush KS Using Six Sigma® methodology to improve handoff communication in high-risk patients. In: Henriksen K Battles JB Keyes MA, editors. Advances in Patient Safety: New Directions and Alternative Approaches (Vol. 3: Performance and Tools). Rockville, MD: Agency for Healthcare Research and Quality (US) (2008). p. 2. Available online at: http://www.ncbi.nlm.nih.gov/books/NBK43658/ (accessed October 5, 2012).

[32] Craig R Moxey L Young D Spenceley NS Davidson MG. Strengthening handover communication in pediatric cardiac intensive care. Paediatr Anaesth. (2012) 22(4):393–9. 10.1111/j.1460-9592.2011.03758.x 22211700

[33] Joy BF Elliott E Hardy C Sullivan C Backer CL Kane JM. Standardized multidisciplinary protocol improves handover of cardiac surgery patients to the intensive care unit. Pediatr Crit Care Med. (2011) 12(3):304–8. 10.1097/PCC.0b013e3181fe25a1 21057370

