# Supplementary figures and images for: Improved patient safety with a simplified operating room to pediatric intensive care unit handover tool (PATHQS)
Source: Front Pediatr. 2024 Jan 24;12:1327381. doi: 10.3389/fped.2024.1327381 (PMC10847360; doi:10.3389/fped.2024.1327381)

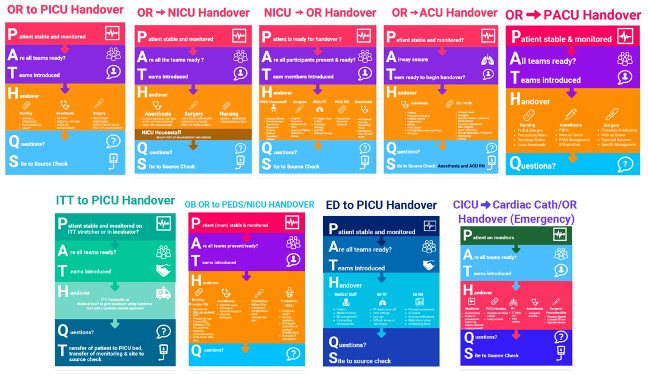

Supplement: Supplementary file 1 [file Image1.jpeg]
